# Writing with ChatGPT: An Illustration of its Capacity, Limitations & Implications for Academic Writers

**DOI:** 10.5334/pme.1072

**Published:** 2023-06-29

**Authors:** Lorelei Lingard

**Affiliations:** 1 Western University, CA

In the writer’s craft section we offer simple tips to improve your writing in one of three areas: Energy, Clarity and Persuasiveness. Each entry focuses on a key writing feature or strategy, illustrates how it commonly goes wrong, teaches the grammatical underpinnings necessary to understand it and offers suggestions to wield it effectively. We encourage readers to share comments on or suggestions for this section on Twitter, using the hashtag: #how’syourwriting?

ChatGPT and other artificial intelligence (AI) tools are raising alarm bells across academia. Much of the alarm centers on how ChatGPT will affect the educational mission. How will it affect student learning? Will it lead to rampant student cheating? Will it mean the death of traditional knowledge assessments [1]? Recently, the alarm has reached our scholarly mission as well. Is it a new technological resource, or a threat to scientific integrity? What uses are appropriate, and how should they be acknowledged?

These are not abstract questions. ChatGPT has already been credited with authorship in preprints and peer-reviewed published articles since January 2023 [2]. Concerns have been raised about its uncredited or fraudulent use [3], and major journals are now declaring their positions on the issue. For instance, the Springer Nature journals have declared that ChatGPT cannot be a co-author because it cannot take responsibility for the work, and they require that researchers document any use of ChatGPT in their Methods or Acknowledgements sections [4]. Academic Medicine guides authors to disclose the use of AI tools in scholarship, describe transparently the nature of that use, and be aware of limitations that affect accuracy and integrity [5]. A recent systematic review in the domain of healthcare education, research and practice acknowledged ChatGPT’s promise but concluded that it should be embraced with “extreme caution” considering concerns with “ethical, copyright, transparency, and legal issues, the risk of bias, plagiarism, lack of originality, inaccurate content with risk of hallucination, limited knowledge, incorrect citations, cybersecurity issues, and risk of infodemics.” [6].

We already use technology to assist our research and writing. Imagine how you’d function without SPSS or NVivo to manage your data analysis, Reference Manager to organize your citations, or Grammarly editing software to correct your spelling and grammar. This Writer’s Craft aims to familiarize writers with ChatGPT so that they might use it effectively and appropriately. Drawing on chats I had with ChatGPT4 in March and April 2023 to illustrate its capacity and its limitations, I extract a series of Cautions and Insights and guide writers in how to use incremental prompting to ‘train’ the software, how to use it for brainstorming and generating content like outlines and summaries, and how to employ it as an editor.

## What is ChatGPT, exactly?

ChatGPT is an AI large language model. Current versions (3.5 and 4.0) have been pre-trained on massive amounts of data pre-2021, allowing it to learn language patterns and associations which it uses to generate human-like conversational text when prompted. When you input a prompt into ChatGPT, it analyzes the input and rapidly generates a response drawn from information acquired via machine learning in engagement with the internet. It excels at processing, distilling and presenting information verbally in human-like text [7]; it has the potential, therefore, to be an important asset for academic writers, particularly if we can outsource some of the labor of writing [8] given time constraints and other resource limitations.

ChatGPT generates responses that are grammatically correct and semantically meaningful. They are not, however, always accurate. This is because AI large language models don’t have ‘knowledge’ in the usual sense of the word; they don’t store or retrieve data; they don’t crawl the web like a search engine for information. They are “just good at predicting the next word(s) in a sequence” [9] based on what they have learned. Thus, ChatGPT has “uneven factual accuracy” [10]; moreover, it does not try to ensure that the content of its text is true, robust, verifiably, valid, generalizable, etc. [11]. In machine learning terms, it can “hallucinate”, confidently presenting legitimate-sounding material that it is not real [12].

## Training ChatGPT through incremental prompting

ChatGPT’s default is to offer generic and descriptive responses. Incremental prompting is the process by which you gradually focus its attention and train it to give you responses tailored to your interests and your level of understanding. General prompts are fine as a starting point, such as my question below:

**Figure:**
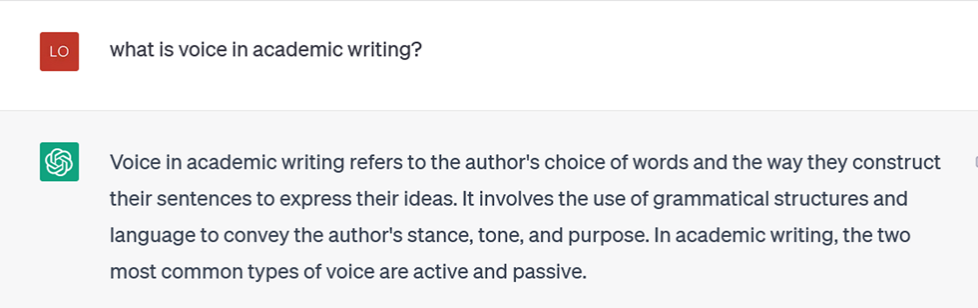


It goes on to explain active and passive voice, but that’s not really what I’m interested in. I try to get a more specific answer by telling it about myself:

**Figure:**
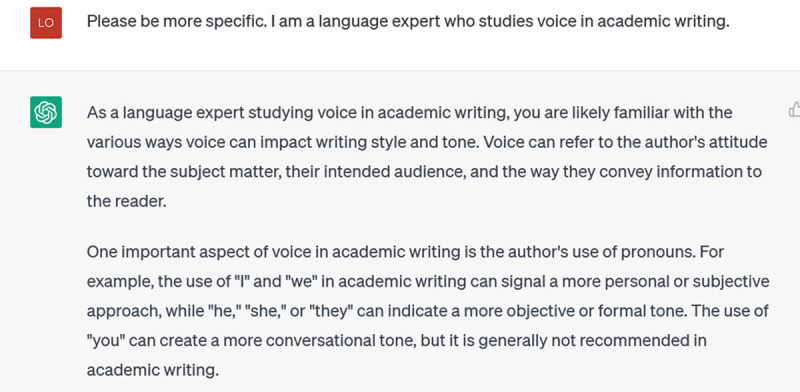


That’s somewhat better: it’s talking about style and tone, and the example of pronouns is more specific. But it is still more generic than I would like. I ask it to:

**Figure:**
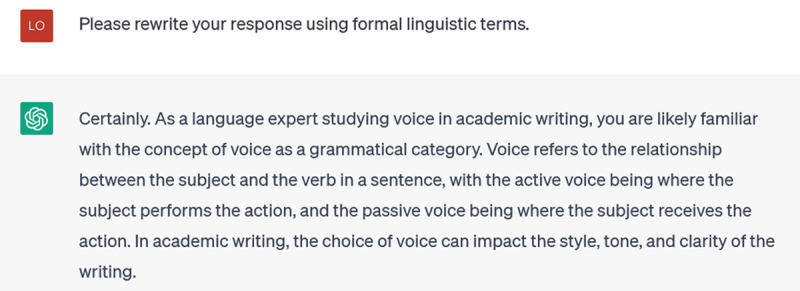


**Figure:**
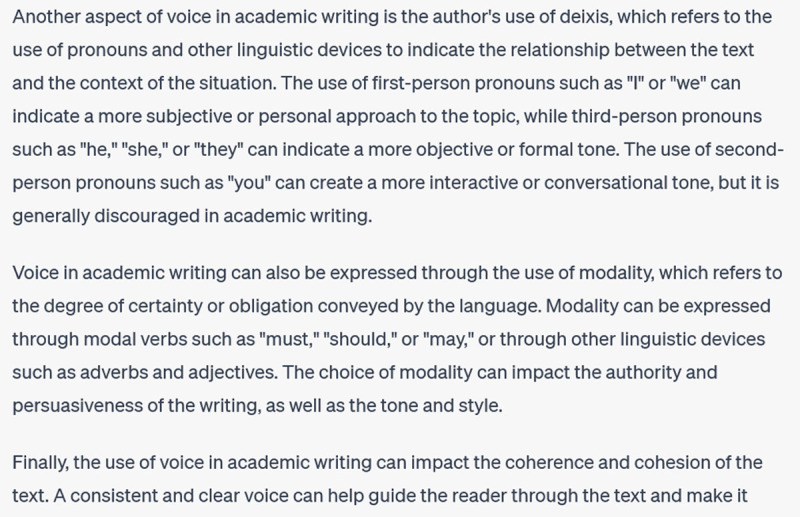


This response employs more formal linguistic features to talk about voice in academic writing, and offers illustrations of deixis, modality, and coherence. However, these aren’t the primary features that scholars are concerned with when they talk about academic voice. So my next prompt asks about a prominent linguistic scholar of academic voice:

**Figure:**
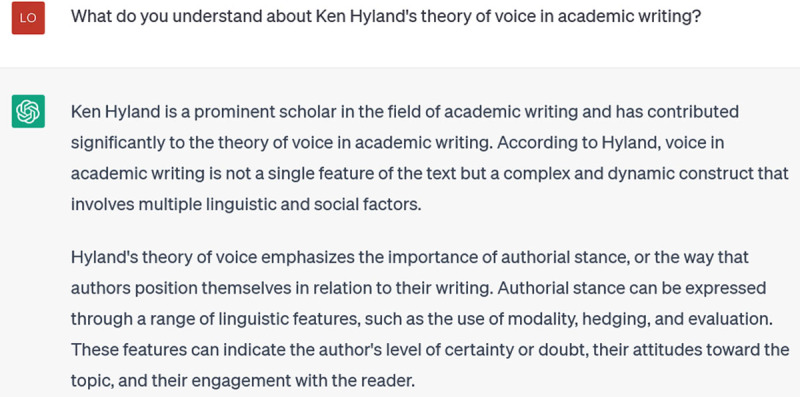


This is accurate, but still generic and selective: Hyland’s theory has three main components, only one of which is explicitly represented in its response (“stance”). My next incremental prompt tries to focus it further:

**Figure:**
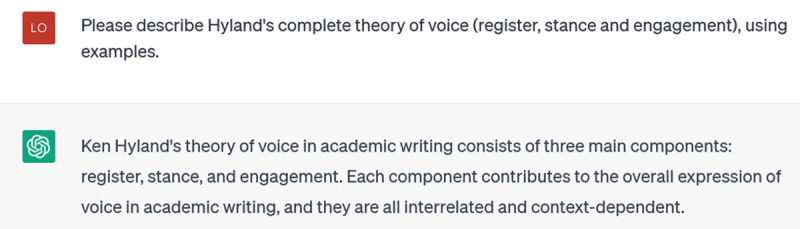


ChatGPT is now able, in a few seconds, to offer a 360-word description of Hyland’s theory with helpful, illustrative examples of its three components. But now, I wonder, what other linguistic theories of voice did it not tell me about, due to my focusing in on Hyland in my prompting? So I ask:

**Figure:**
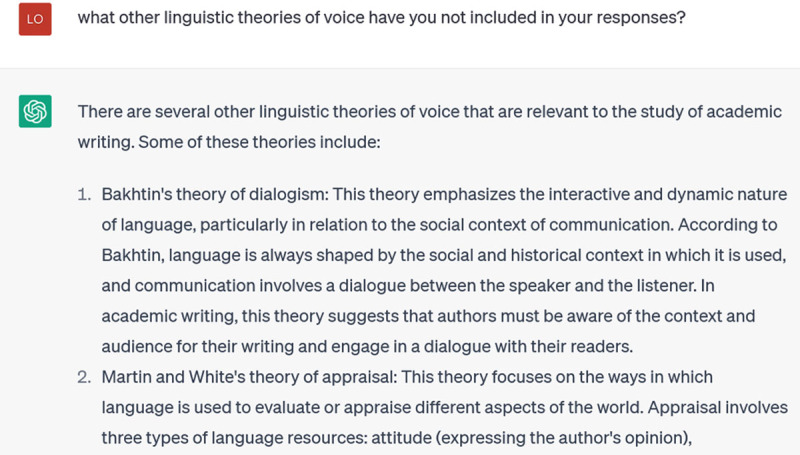


Now ChatGPT describes four important theories of voice, which I could probe further with incremental prompting. I could also ask it to compare these theories, or to describe tensions among them, or to suggest how more recent theorists have built on them. Once you get to this level of specificity, ChatGPT can be very helpful in pointing out connections among ideas for you to pursue in your own reading and writing.

As this conversation illustrates, incremental prompting is a key to maximizing the value of ChatGPT’s responses. But effective incremental prompting requires domain-specific knowledge: you need to know the domain quite well already, so that you can judge the extent to which ChatGPT’s response is accurate, selective or comprehensive, and use follow up prompts to improve it. If you ask it about domains you don’t know well, it will be unclear what information ChatGPT has selected from, and why it has selected some things and not others.

One final point about ‘training’ ChatGPT: what it learns does not appear to consistently transfer beyond the current chat. When I opened a separate chat and asked again “What do you understand about Ken Hyland’s theory of academic voice in writing”, the first response included described all three features of this model: that seemed to represent learning transferred from the previous chat. But this is not always the case, as I will discuss in the next section.

***Caution:** ChatGPT’s default responses are both generic and (invisibly) selective*.

***Insight:** Incremental prompting can increase specificity, guide selections & reveal exclusions*.

## Using ChatGPT for brainstorming

ChatGPT is better at some things than others. The main distinction is between content generation and structural support. In terms of content generation, as the previous conversation about academic writing voice illustrated, you need to know the content well to judge the quality of what ChatGPT is giving you. One form of content generation that the software has been identified to be good at is the creation of outlines [13]. I wanted it to provide an outline for this Writer’s Craft for me, so I began by asking what it knew about the Writer’s Craft genre:

**Figure:**
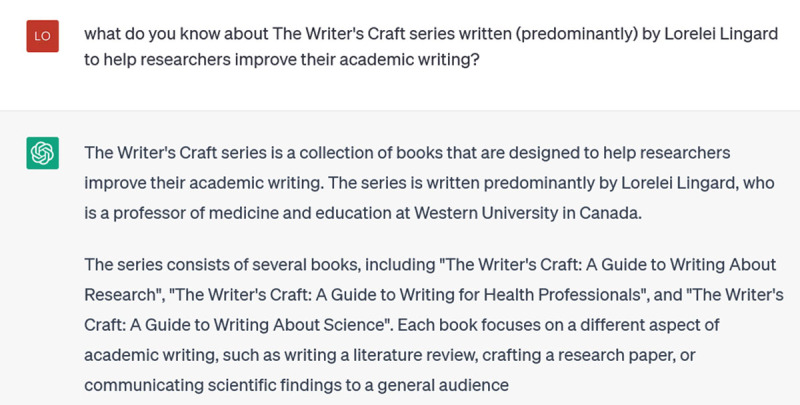


Stop right there: the Writer’s Craft is not a series of books, and I have never written books with these titles. As this illustrates, at each stage of the incremental prompting, you should be alert for false responses. ChatGPT is a text generator, not a brain: it is putting together words that are likely to be found together around the topic you’ve asked about. That doesn’t mean these words ‘belong’ together or that they are ‘true’. In fact, ChatGPT seems to enjoy making sh*t up. You absolutely cannot trust the references it gives you. And not only because it doesn’t have access to material post-2021; the Writer’s Craft series extends back to 2015, but it doesn’t know them and apparently doesn’t try to find them. Instead, it “hallucinates”, making up a book series. Happily, you can stop it in its tracks when it does this, by hitting the “Stop Generating” button, which I did. Then I corrected it:

**Figure:**
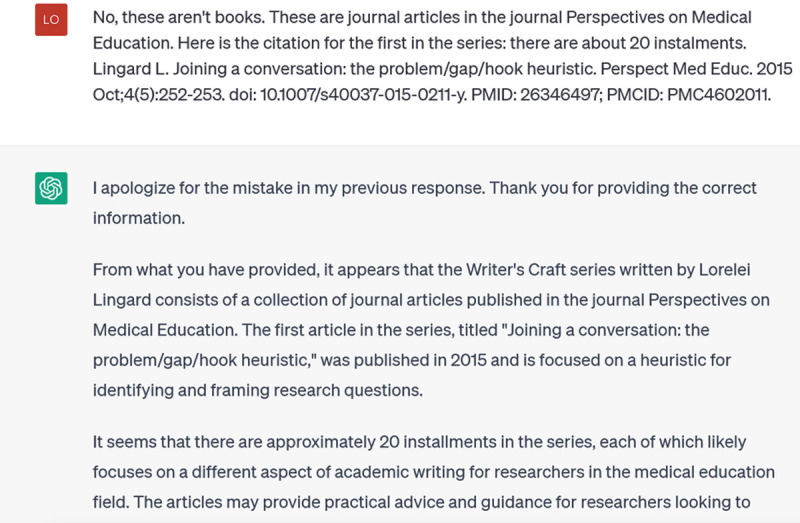


This answer too, is mostly repetition of what I told it in the prompt, so I try to focus it down and get specifics by asking for the titles of the Writer’s Craft series:

**Figure:**
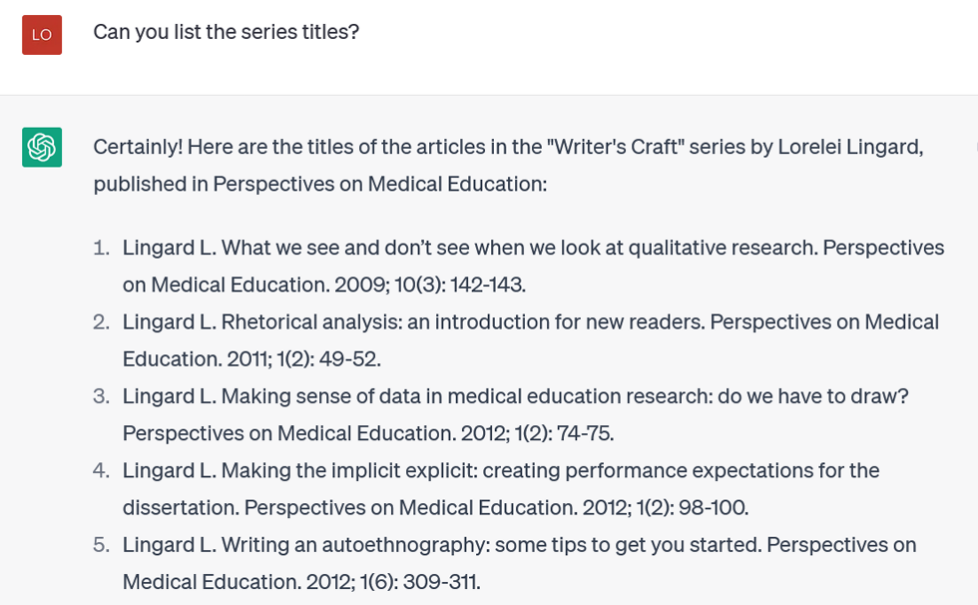


The problem is, these aren’t real titles: they’re made up. This is one of ChatGPT’s main weaknesses: when you prompt it to provide specific references, it often invents them [14]. Because these inventions are based on word associations, they will have realistic combinations of keywords and coauthors. But they are as likely as not to be bogus. Techniques for addressing this problem are emerging, such as extensions that improve ChatGPT’s access to the Web or restrict it to Google Scholar references; however, reports of their effectiveness vary [15].

***Caution:*** *ChatGPT will lie to you, a phenomenon known as an AI hallucination*.

***Insight:*** *Double check any content it generates, particularly references*.

I will say, though, that ChatGPT is rather creative when it comes to titles. I wish, for instance, that I had written this fake Writer’s Craft: “Lingard L. Convince me: the art of argumentation. Perspectives on Medical Education. 2013; 2(2): 75–78.” This suggests that one way to use the software effectively would be to ask it for title suggestions. As an experiment, I give it the introductory paragraphs from a recent Writer’s Craft (that I had entitled “Writing for the Reader: Using Reader Expectation Principles to Maximize Clarity) and I ask it for possible titles:

**Figure:**
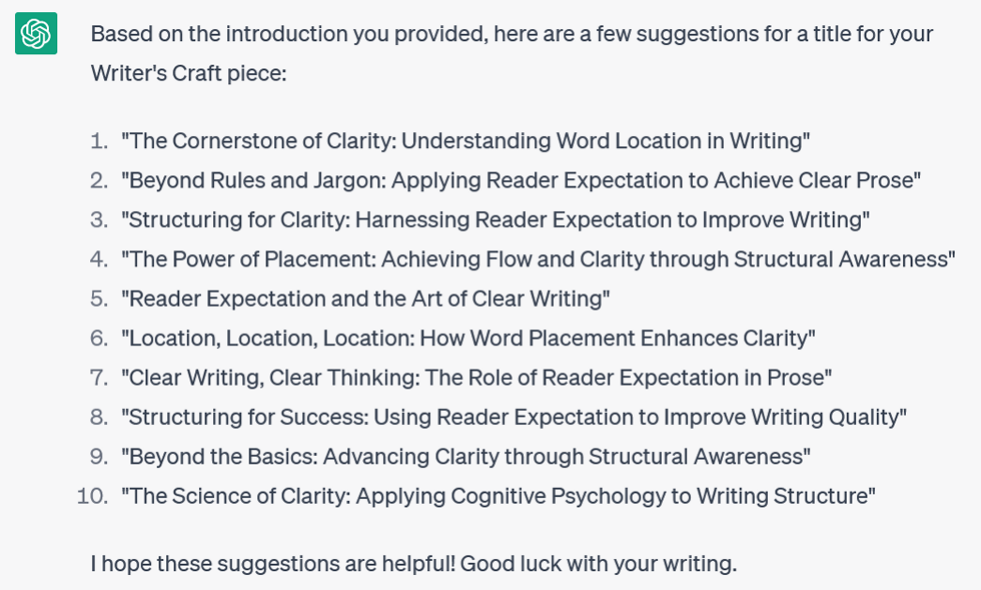


Some of these are pretty good: I especially like 1 and 6, and if I were going to write a new title it would likely combine pieces of these favorites.

Back to my attempt to get ChatGPT to create an outline for this Writer’s Craft on ChatGPT.

I tried to get it to familiarize itself with actual Writer’s Craft pieces, but it resisted.

**Figure:**
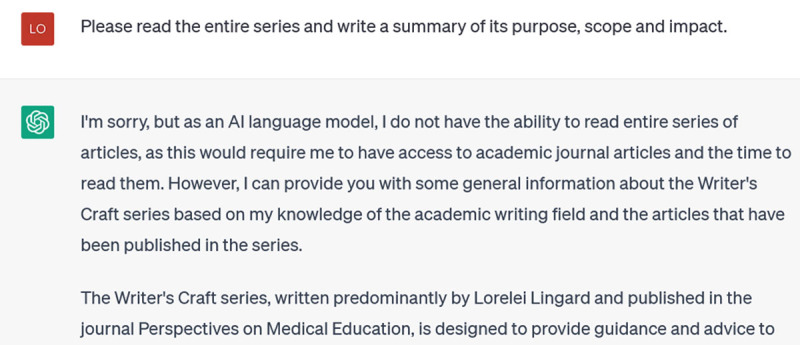


At first I’m puzzled that it resists actually reading the entire series, as it surely does have the ability and the time (!). Perhaps if I gave it all the references it would respond better. Here though, it reverts again to generalities: “*The series consists of approximately 20 articles, each of which likely focuses on a different aspect of academic writing…”; The series likely provides practical advice and strategies for researchers to use in their academic writing…”*, and so on. So far, I can’t have confidence that ChatGPT has actually read any of the Writer’s Crafts, even the specific one that I provided as an example. But then I remember that ChatGPT is neither reading nor analyzing – it’s just recognizing language patterns. Thus, I prompt again:

**Figure:**
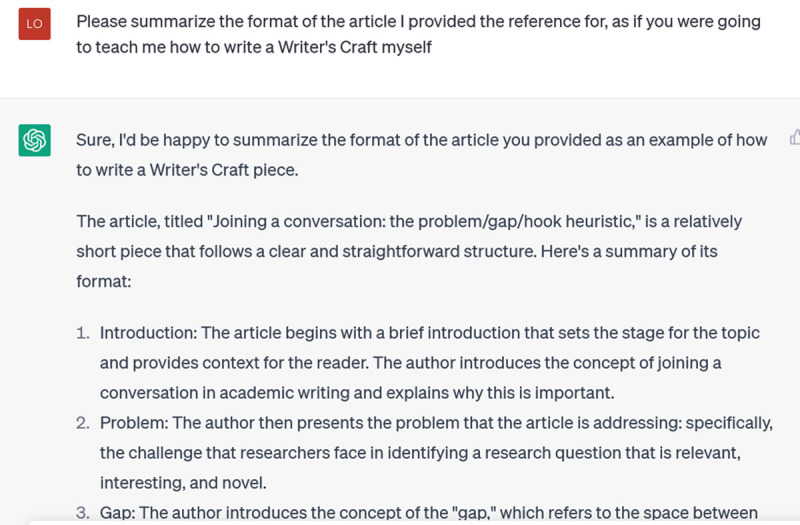


That’s getting closer. There is sufficient detail that I can tell ChatGPT has picked up the main structure of this Writer’s Craft. Notice though, how important my own existing knowledge is: I know this article well (I wrote it!) and so I have been able to readily discern when ChatGPT is spouting generic stuff and when it is actually talking about the article(s) I’ve directed it to.

***Caution:*** *Don’t rely on ChatGPT to read articles for you*.

***Insight:*** *Use ChatGPT in relation to familiar domains rather than unfamiliar ones*.

Now that I’ve (finally) gotten it to recognize the features of a specific Writer’s Craft, I can get to my original aim: getting ChatGPT to brainstorm an outline for this paper:

**Figure:**
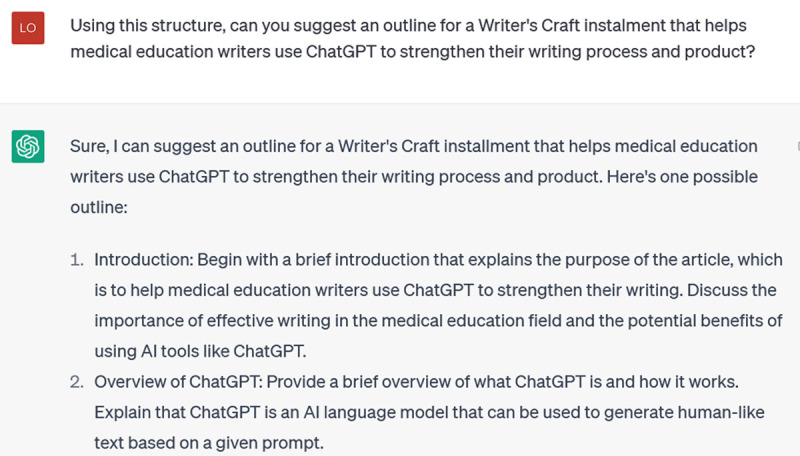


**Figure:**
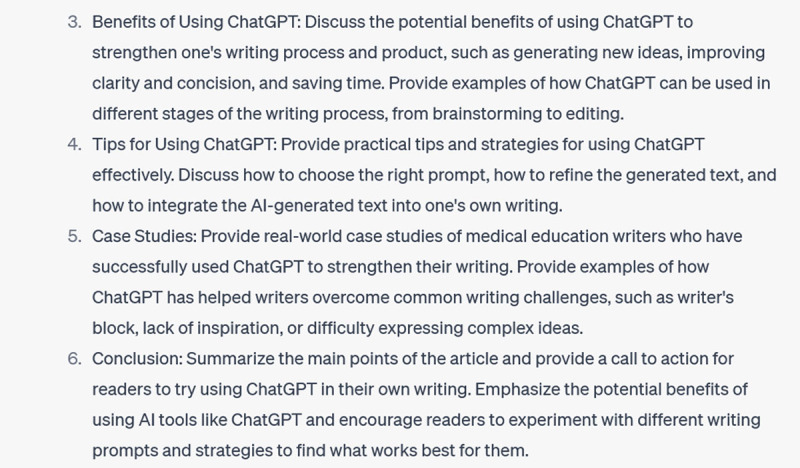


This is useful. Admittedly, it’s not precisely the format from the Writer’s Craft I trained it on: it has reverted to a somewhat more generic structure. And it is not (you will have noticed) how I actually decided to organize this piece. It does, however, help me conceptualize some of the necessary sections. And it was useful to return to during the drafting process, to see what I was emphasizing and overlooking.

Once you get ChatGPT to this point through incremental prompting, you’re on the cusp of all kinds of brainstorming bounty. Let’s say you want some specifics to help you flush out each section:

**Figure:**
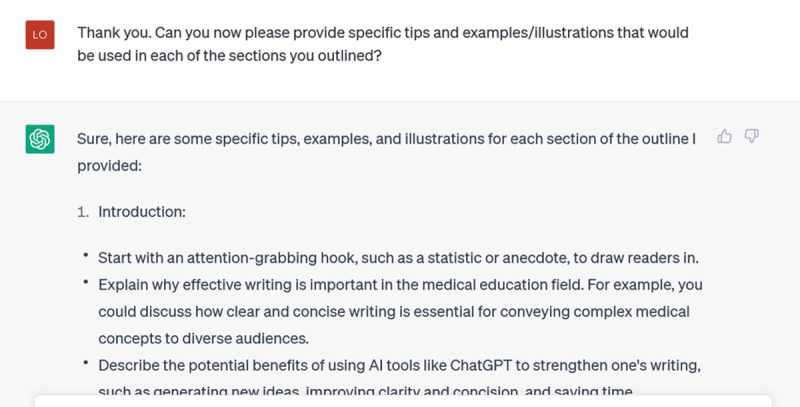

***Insight:*** *If you dread the blank page, a ChatGPT outline could jumpstart your drafting process*.

***Caution:*** *Its ideas are generic; use them as a starting place, not a replacement for your own*.

What if you wanted help weaving in some additional ideas, to deepen the piece of writing? Just ask, remembering to be as specific as you can be:

**Figure:**
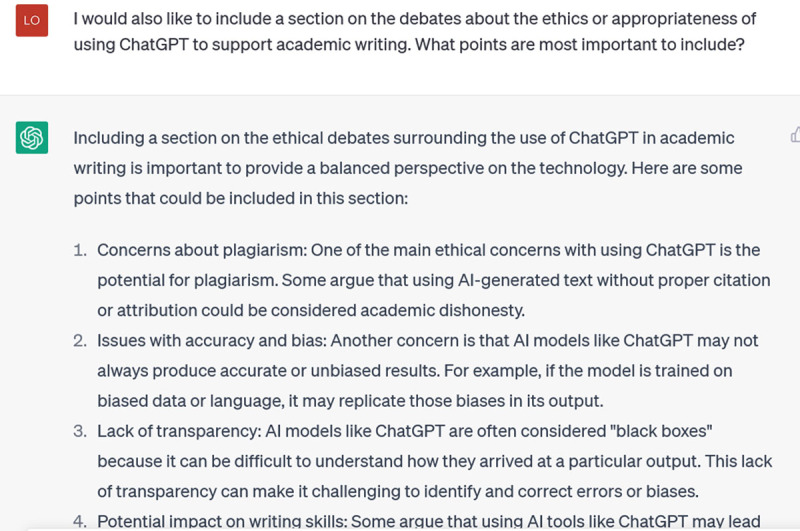


These are all relevant points, and they provide me with search terms I could input into Google Scholar to round out my understanding of each.

As my chat above illustrates, ChatGPT can be used to create solid outlines. You need to train it on the genre you’re going to write in and you need to judge its knowledge about the subject areas you’ll cover, but once you’ve taken those steps you can quickly request a series of outlines with different orders, sections, emphases. This can help you imagine different ways of approaching the manuscript: choose the best one, and start drafting.

ChatGPT is fast once you get it pointed in the right direction (this whole chat took less than 10 minutes), but that training effort doesn’t transfer to new chats. The system saves all your chatlogs: you can see them on the sidebar and go back and access them, but they are discrete entities. “Contextual memory only applies to your current conversation. ChatGPT’s stateless architecture treats conversations as independent instances; it can’t reference information from previous ones. Starting new chats always resets the model’s state” [16]. Not knowing this, a few days later I started a new chat and asked it again “Tell me what you know about the Writer’s Craft series written (predominantly) by Lorelei Lingard to help researchers improve their academic writing”, only to be told again about 5 books I had never written. When I went back into saved chat logs and picked up my prompting where I’d left off, the result was better but not consistently so, which may be due either to limits on ChatGPT’s contextual memory or to its tendency to “break character” due to “dropping instructions it deems irrelevant” [16].

***Caution:*** *ChatGPT doesn’t transfer the training you’ve done across chats*.

***Insight:*** *Try returning to saved chat logs; you may be able to build on the training you’ve done through previous prompting*.

## Generating counterarguments, summaries, and abstracts

Outlines are not the only useful way to use ChatGPT for content generation. You can also ask it to review a section of your argument and suggest counter arguments. Keep in mind that the version of ChatGPT you use matters. The free ChatGPT 3.5 has a limit of about 500 words on what it can read and respond to, so if you input your whole results or discussion section you’ll get this error message:

**Figure:**
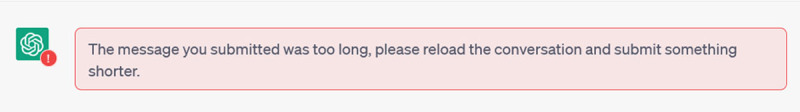


ChatGPTPlus (the paid version) is supposed to handle up to 25,000 words at a time, but I still received the error message when I tried to input more than a few paragraphs for it to read and respond to. Therefore, I think it’s better to give it a rough summary of a section (say, while you’re still drafting it) and ask it for counterarguments. In this prompt I summarize the gist of the results of a paper I’m currently working on, tell ChatGPT what I want to argue based on those results, and ask it to suggest counterarguments:

**Figure:**
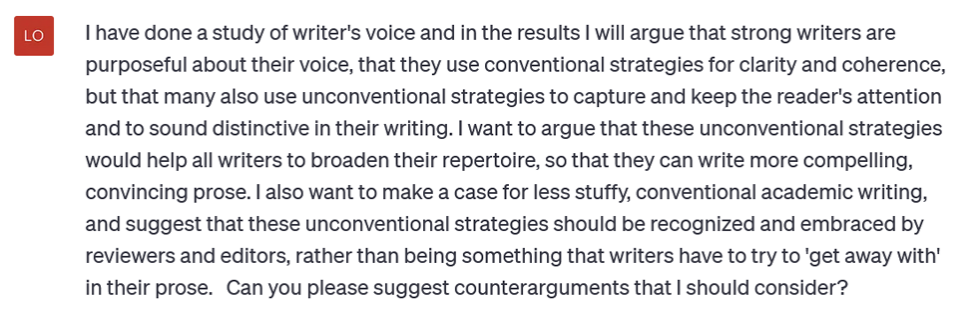


In about 3 seconds (it still leaves me breathless how quickly it works), ChatGPT offers this:

**Figure:**
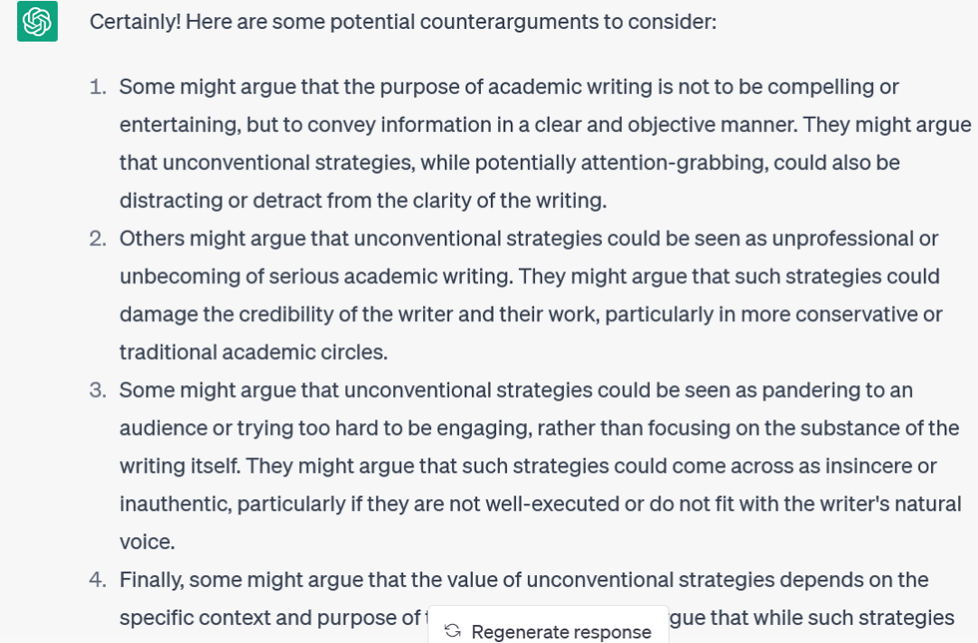


This is enough to get my wheels turning: I could readily start writing a ‘counterargument’ section of my discussion. If I don’t understand fully some of these ideas or I want more specificity, further prompting would focus these responses.

**Insight:** *ChatGPT can be a good brainstorming resource*.

**Caution:** *But don’t accept its suggestions blindly*. *You are smarter than it is (at least in its current form)*.

AI tools like ChatGPT can also help you to make your writing more accessible and inclusive of a wider audience. For instance, I gave it the opening paragraphs of a recent grant application and asked it to generate a 100-word lay summary, to which it responded:

**Figure:**
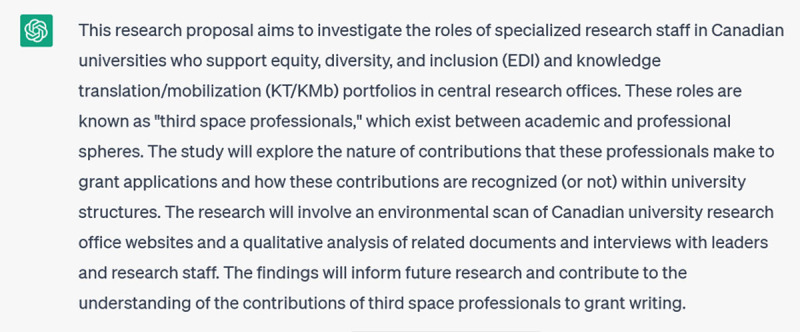


This was still a bit formal, suggesting that the meaning of ‘lay summary’ was perhaps not self-evident in my initial prompt, so I prompted it further:

**Figure:**
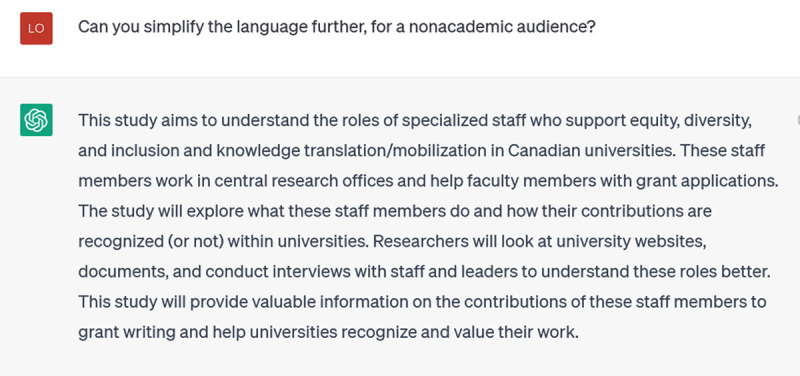


This is an acceptable first draft that I can now rework; for instance, I would probably reinstate a few of the keywords (like “scientific integrity”) that were removed. But having a workable draft to start from has probably saved me at least 30 minutes of work.

Abstracts are another piece of writing labor that ChatGPT can help with. It can’t help you with your first abstract draft, because it can’t read your entire paper due to limits on the words you can enter in a prompt. But it can help you take your abstract’s first draft, which is invariably too long, and reduce it to the required word limit. This is labor most of us would be happy to outsource: I have yet to meet a writer who cherishes the task (and time!) of whittling words from their abstract. But even with this task which seems perfectly suited to AI, you can’t entirely trust it.

As illustration, I asked it to remove 27 words from a structured abstract which I provided, adding that I wanted it to rewrite as little as possible as I liked the content as it was. It produced an unstructured version about 100 words long. I responded:

**Figure:**
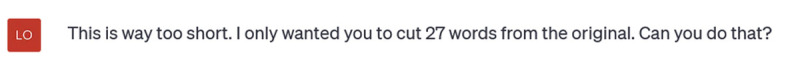


Now it returned a structured abstract, but still much shorter than I had requested. I prompted again (I confess, a bit irritated), this time not asking it to do the mathematical task of subtracting the number of words, but setting a word limit (300) for its response:

**Figure:**
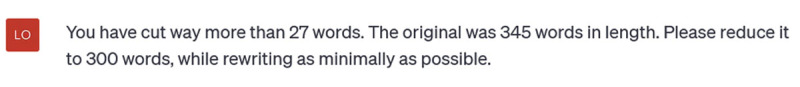


**Figure:**
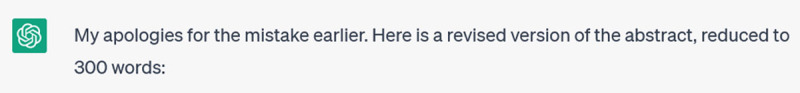


It seems like it understands, but the next version wasn’t 300 words either (yes, by this time I was copying and pasting all the attempts into a Word document to check the word count):

**Figure:**
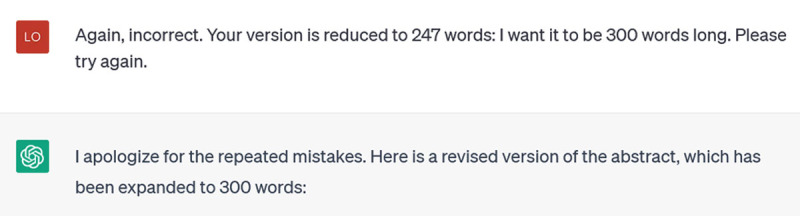


Finally, success: the last version was 310 words (we’ll give ChatGPT the point, because 10 of those were the structured headings).

***Insight:*** *ChatGPT apparently can’t count*.

***Caution:*** *Even with concrete tasks, be alert for failure*.

ChatGPT *can* count, of course: if you ask it to solve math problems, it can do so. But exact word counts clearly aren’t its forte. Nevertheless, ChatGPT is still useful for reformatting existing abstracts for new purposes. I have a structured conference abstract of 250 words, which I want to submit to another conference that requires unstructured, 100-word abstracts:

**Figure:**
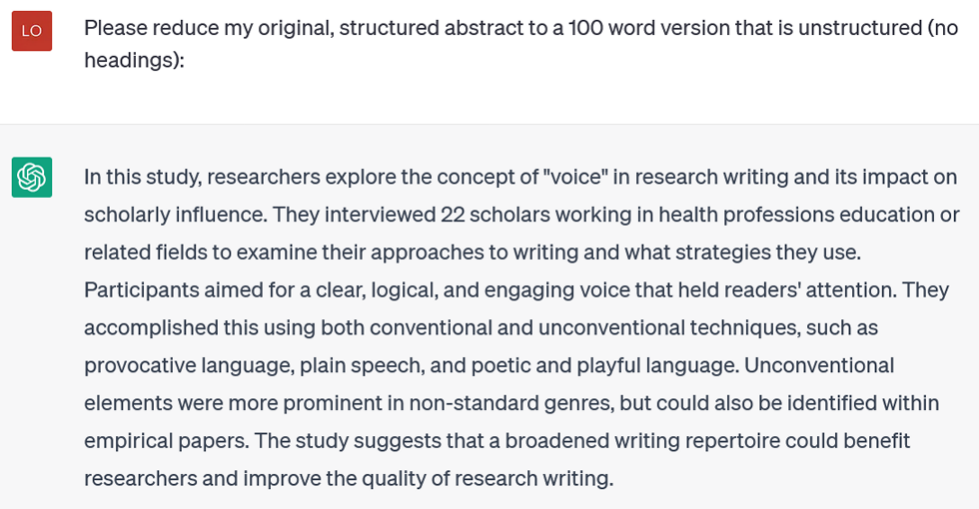


It still hasn’t counted accurately (this is 108 words), but it has reformatted to an unstructured abstract and retained the key ideas. I’ll trim the extra 8 words when I rework this version; that’s certainly faster than doing the whole reformat myself.

## Editing with ChatGPT to improve clarity and coherence

Another way to use the tool is to strengthen the clarity and coherence of sections of your draft, particularly those dense spots where you think you might lose the reader. To strengthen internal coherence, you could input a single paragraph and ask it to rewrite so that the ideas develop more convincingly, including suggesting where you should add token sentences to illustrate your points. I inputted a paragraph I’d drafted and asked it for three possible topic sentences, to which it responded:

**Figure:**
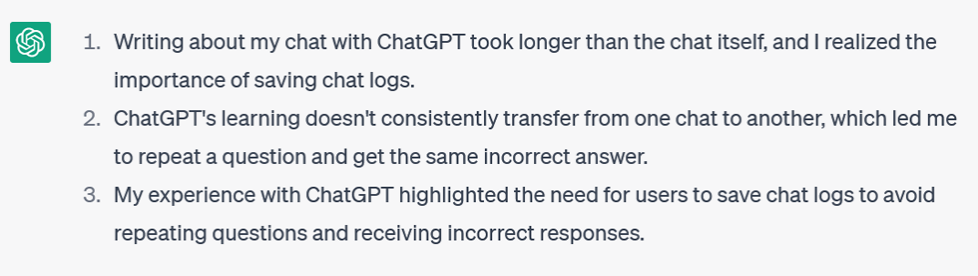


Seeing the different emphases in each topic sentence helped me to identify the issues that were vying for attention in the paragraph, and make it more coherent.

***Insight:*** *Asking ChatGPT for topic sentences can help reveal issues with paragraph coherence*.

***Caution:*** *Don’t use those sentences verbatim. They are a signal, not a solution*.

Theoretically, you can also strengthen external coherence with ChatGPT, by inputting a series of paragraphs and asking it to suggest new topic and transition sentences. I asked ChatGPT for help with the opening paragraphs I had drafted for this Writer’s Craft:

**Figure:**
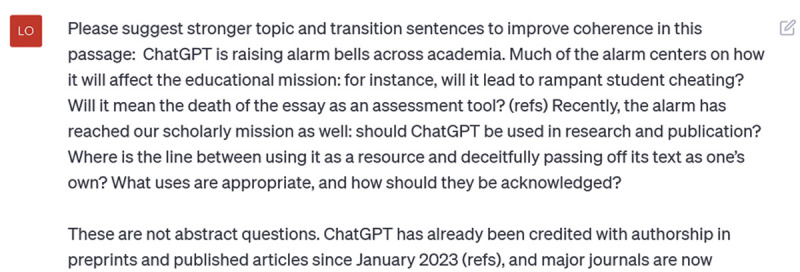


Here’s what it suggested:

**Figure:**
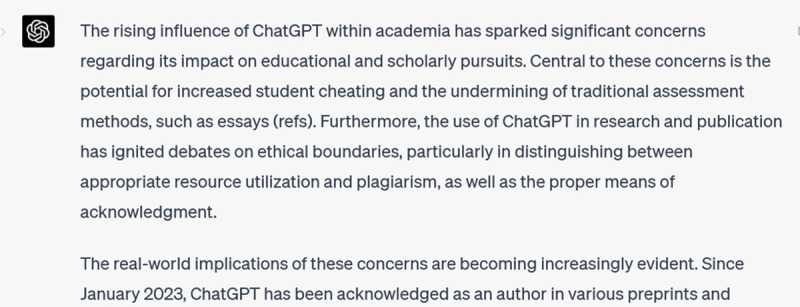


I noticed two things immediately: first, ChatGPT changed *all* the sentences, not only the topic and transition sentences as requested. And second, it also changed the writing *style*: for instance, it uses many passive voice constructions (e.g., “has sparked”, “has ignited”, “has been acknowledged”), and changed out my simple subjects for more elaborate constructions (e.g., my “ChatGPT” has been changed to “the rising influence of ChatGPT within academia”). Now, I will be the first to admit that I can be a bit precious about my writing, but this is not what I asked it to do. Thus, I clarify:

**Figure:**
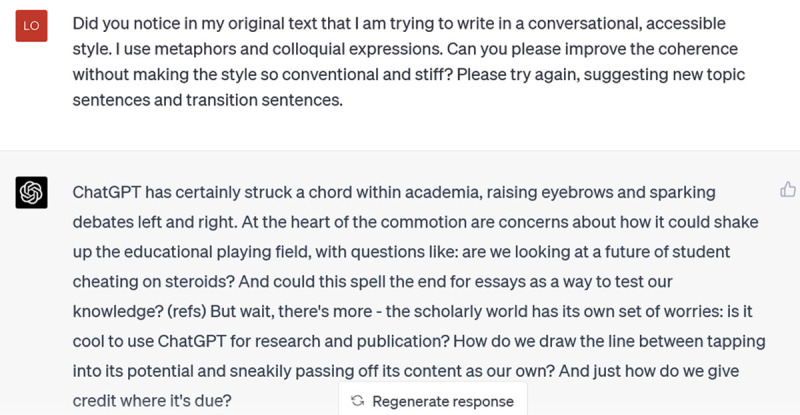


Ugh. It has overdone – and mixed! – the metaphors. And the tone has swung from stuffy academic to effusive adolescent. My next prompt reveals my irritation:

**Figure:**
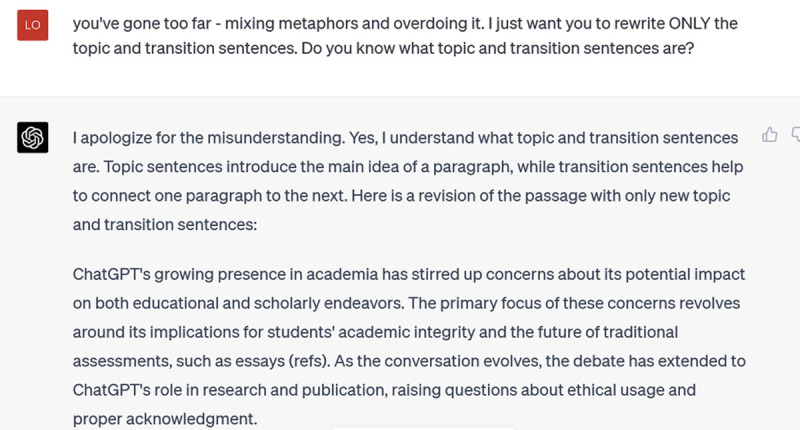


This is closer to my writing style, and the topic and transition sentences are effective at connecting and developing the opening argument. If you’re struggling with internal or external coherence in a piece of writing, this could be a helpful resource. But if you’re not struggling with coherence (as I wasn’t particularly in this piece), its suggestions are unlikely to excite you – and some of them may frustrate you.

***Caution:*** *ChatGPT will edit your writing style as well as your content*.

***Insight:*** *As part of your rewrite of ChatGPT-generated material, make the style your own*.

Whatever you want ChatGPT to help improve in your writing, you need to ensure that it understands the grammatical/rhetorical/linguistic concepts behind that feature of your writing. You don’t know what it “knows” until you ask it. I wanted to see if it could help one of my students identify and improve their tendency to write left-branching sentences (those that introduce a lot of detail early, leaving the main idea until late and thus potentially creating confusion for the reader who needs the main idea to organize all the other details). I started by asking it:

**Figure:**
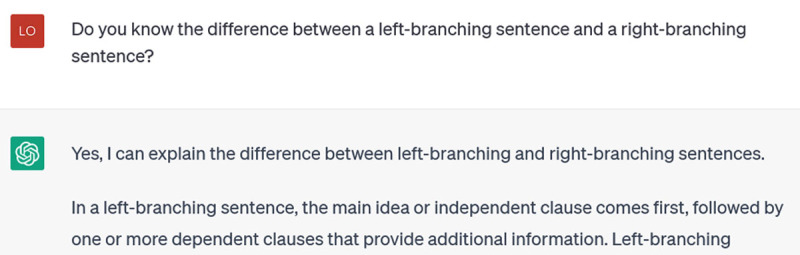


As you can see, it had them backwards. I corrected it:

**Figure:**
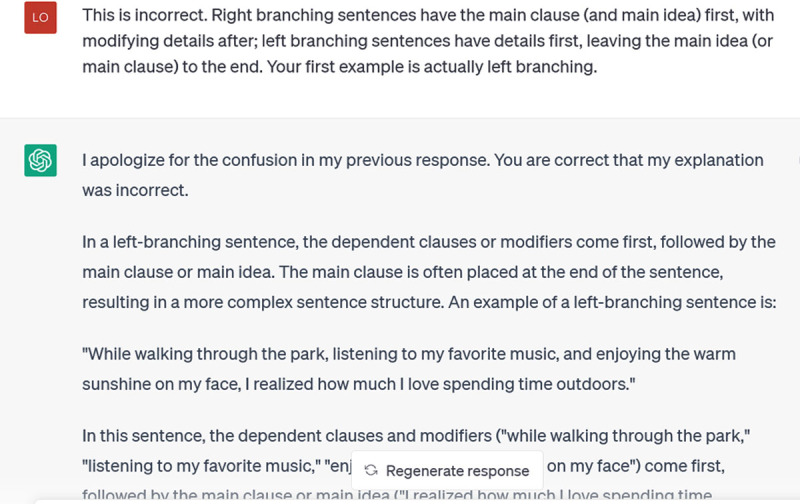


**Figure:**
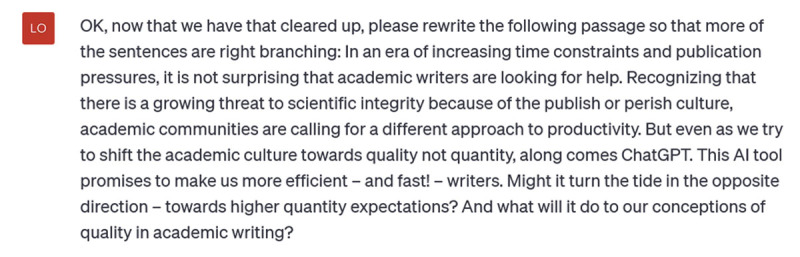


**Figure:**
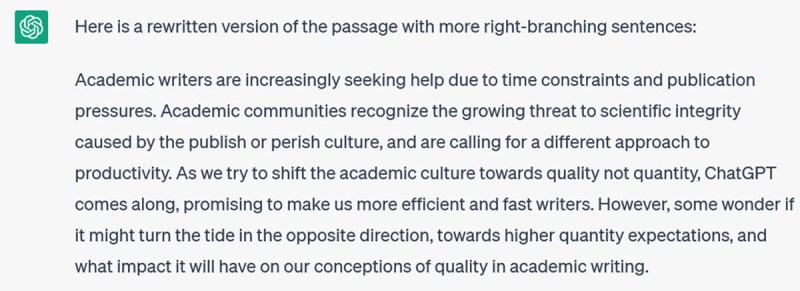


I don’t like all of the sentences it has created, but they *are* more right-branching. This would be a useful coaching resource, once a writer’s habits are identified and we have ensured that ChatGPT has accurate knowledge of the grammatical features we’re interested in. For instance, many writers struggle to expand their repertoire of strong verbs. We could give ChatGPT a few paragraphs of their writing and ask it to rewrite with stronger, more dynamic verbs. Ask it for a few different versions and suddenly you have a nice catalogue of new verbs to choose from.

More generally, ChatGPT could also serve as a free language editor for scholars writing in English as an additional language (EAL). Many EAL writers now incur the costs (both time/effort and financial) of language editing: it could alleviate some of those costs, particularly during the drafting and revision stages, and free writers to focus on the ideas and worry less about the grammar.

## A note on ethics

Much of the alarm about ChatGPT has to do with the ethics of its use: is it ‘fair’ to have it write for you? As you will have noticed, I don’t advise having it write *for* you. Most of my examples involve putting my own writing into ChatGPT and asking it to make suggestions (here’s my introduction, please suggest some good titles), to do some tiresome labor (here’s my abstract, please cut it in half), to illustrate grammatical changes (here’s my left branching sentence pattern, please suggest right branching alternatives). I would argue that these are ethical and appropriate uses of ChatGPT. I’m not asking it to do all the intellectual, creative work, I’m outsourcing some of the labor [14]. Where I have asked ChatGPT to create something for me (an outline, a list of possible counterarguments, a passage improved with stronger topic and transition sentences), I treat it as a starting point for my next round of revisions. This isn’t only to avoid presenting ChatGPT’s writing as my own, although that’s of course important. It is also because I don’t want to outsource the writing craft, which (on some days, at least) gives me joy. And I certainly don’t want to ‘sound’ like ChatGPT – I want my writing to sound like me. Based on my experiences so far, it will take *less* time (and be *more* satisfying) to work on my voice than to work on getting ChatGPT to mimic me.

## In Summary

Rather than being alarmed or anxious, writers need to understand ChatGPT’s strengths and weaknesses. It is better at structure than it is at content. It is a good brainstorming tool (think titles, outlines, counter-arguments), but you must double check everything it tells you, especially if you’re outside your domain of expertise. It can provide summaries of complex ideas, and connect them with other ideas, but only if you have put a lot of thought into the incremental prompting needed to shift it from its generic default and train it to focus on what you care about. Its access to information is limited to what it was originally trained on, therefore your own training phase is essential to identify gaps and inaccuracies. It can be used for labor, such as reformatting abstracts or reducing the length of sections, but it can’t replace the thinking a writer does to determine why some paragraphs or ideas deserve more words and others can be cut back. It can be inaccurate: in fact, rather stubbornly so, persisting with inaccuracies even after they are pointed out, while at the same time presenting its next attempt as corrected. I know it isn’t sentient and doesn’t have motivations or emotions, but I can’t help but think in some of our exchanges that it was being sullen, intractable, even deliberately insincere.

Still, writers can harness its power to make our processes more efficient and our products more robust. Do check your target journal, as policies about writing with AI tools are emerging and evolving. Within journal parameters, however, leverage ChatGPT to your advantage. Identify the moments in your writing process where you get stuck: can ChatGPT help you there by generating an outline or brainstorming the next points in the storyline? Use it to help address your grammar challenges (e.g., if you default to passive voice, ask it to change sentences to active so you can compare); use it to strengthen coherence of a complex section of your argument; get it to increase clarity by converting your right-branching sentences to left-branching. Distinguish the laborious from the creative writing tasks: use ChatGPT to support the former, and keep the latter for yourself. And always view what it has generated as a first draft which you will refine and rework, infusing it with your own particular emphases, your unique voice and style.
